# Thinking of future as an older individual increases perceived risks for age‐related diseases but not for COVID‐19

**DOI:** 10.1002/ijop.12789

**Published:** 2021-06-24

**Authors:** Dario Monzani, Marco Marinucci, Luca Pancani, Patrice Rusconi, Davide Mazzoni, Gabriella Pravettoni

**Affiliations:** ^1^ Department of Oncology and Hemato‐Oncology University of Milan Milan Italy; ^2^ Applied Research Division for Cognitive and Psychological Science, IEO European Institute of Oncology IRCCS Milan Italy; ^3^ Department of Psychology University of Milano – Bicocca Milan Italy; ^4^ School of Psychology University of Surrey Guildford UK; ^5^ Department of Cognitive Sciences, Psychology, Education and Cultural Studies (COSPECS) University of Messina Italy

**Keywords:** Age priming, Age‐related diseases, COVID‐19, Future‐oriented thinking, Risk perception

## Abstract

Actively thinking of one's future as an older individual could increase perceived risk and risk aversion. This could be particularly relevant for COVID‐19, if we consider the common representation of the risk of being infected by COVID‐19 as associated with being older. Increased perceived risk could bear consequences on the adoption of preventive behaviours. Thus, we investigated whether increasing the salience of individuals' future as an older adult would impact on their perceived risk for COVID‐19 and medical conditions varying for age‐relatedness. One hundred and forty‐four Italian adults (*M*_age_ = 27.72, range: 18–56) were randomly assigned to either a future as older adult thinking or control condition. Perceived risk for COVID‐19 and other strongly, and weakly age‐related medical conditions during the lifetime was measured. Results showed that thinking about the future as an older adult increased perceived risk for strongly and weakly age‐related diseases, but not for COVID‐19. The salience of the COVID‐19 outbreak may have raised the perceived risks in both experimental conditions, making the manipulation ineffective. In conclusion, manipulating future‐oriented thinking might be a successful communication strategy to increase people's perceived risk of common diseases, but it might not work for highly salient pathologies such as COVID‐19.

Can focusing on a future time frame influence individuals' risk perception? The social priming literature has shown that actively thinking of one's future as an older adult could increase risk perception and risk aversion. For example, Hershfield et al. (2011) induced thinking about the future as older individuals by presenting people with a digital representation of their age‐morphed future selves versus their current selves in a virtual reality context. Their results demonstrated that people exposed to this manipulation are more likely to be conservative and give greater weight to long‐term savings by allocating more money for their retirement than people presented with their current self. Similarly, asking people to actively think about their future (i.e., “10 years from now”) promotes risk aversion when considering their current preferences for different types of investments (Monroe et al., 2017). Specifically, people thinking about their future preferred less risky financial assets than participants who focused on their present condition. Recent empirical evidence has shown that, when thinking about the future, people generally focus on uncertainty and reflect on what could go wrong. This kind of reflection might in turn foster risk avoidance, caution, and attentional focus on any potential risk (Baumeister et al., 2016; Monroe et al., 2017). However, while these results suggest that actively thinking of one's future as an older individual could increase perceived risk and risk aversion when considering financial contexts, no research has ever established whether this would apply also to health‐related risks, such as COVID‐19 and medical conditions. The present study aimed to fill this gap in the literature, by investigating whether asking individuals to actively focus on their own future as an older individual would affect their evaluation of their perceived risk of incurring during their lives in COVID‐19‐related and other medical conditions as a function of their age relatedness.

## RISK PERCEPTION AND BEHAVIOUR CHANGE

Perceived risk (or perceived likelihood, perceived susceptibility), namely people's subjective evaluation of their risk of a disease or an adverse outcome, is placed as a core construct in many health behaviour models, such as the Protection Motivation Theory, the Health Belief Model, and the Health Action Process Approach (for an overview, see Ferrer & Klein, 2015). Specifically, people with higher perceived risk are more likely to adopt effective preventive measures and healthy behaviours. This mechanism becomes relevant during pandemics: Heightening COVID‐19‐related perceived risk might be crucial in fostering the adoption of healthy and precautionary behaviours. Research on past epidemics (e.g., SARS, H1N1 and Ebola) has shown that risk perception is positively associated with effective precautionary measures including mask‐wearing, hand washing, and disinfecting the home (Bish & Michie, 2010; Bults et al., 2011; Katz et al., 2012; Lau et al., 2010). Regarding the COVID‐19 pandemic, research has demonstrated that increased perceived risk for infection and infection fatality were positively associated with handwashing and keeping social distancing (Bruine de Bruin & Bennett, 2020; Czeisler et al., 2020). Given this empirical evidence, risk perception is often targeted by preventive interventions aimed at changing health behaviours. Indeed, the promotion of people's awareness regarding health consequences and the likelihood of a specific disease is one of the core techniques of behavioural change interventions (Michie et al., 2013). A recent meta‐analysis assessing the efficacy of behavioural change interventions to promote hand hygiene and face mask use to limit the spread of respiratory viruses highlighted that most interventions were specifically designed to target and promote the perceived risk of infection (Perski et al., 2021). Similarly, the relevance of increasing risk perception among those who still do not feel personally threatened by COVID‐19 has been underlined by a recent behavioural science approach enhancing potential interventions to promote adherence to social distancing (West et al., 2020).

## COVID‐19 AND AGEISM

From this point of view, promoting COVID‐19 perceived risk might be crucial for people under 60 since these individuals are more likely to perceive a lower risk of dying from or getting COVID‐19 and report lower adherence to social distancing and handwashing behaviours (Bruine de Bruin, 2020; Carlucci et al., 2020). Objective data on infection rates might have misled young people due to ageism and biased risk perception (Ayalon et al., 2020). Specifically, the association between being older and the higher risk of infection was emphasised worldwide. In many circumstances during the spread of the pandemic, medical and institutional sources of information conveyed the idea that COVID‐19 mostly concerned older people (Petretto & Pili, 2020). Also the mass media conveyed the view that older people, namely individuals over 60 are more at risk of being infected and dying from the virus than young people (Ayalon et al., 2020). The main reason for this message was the higher COVID‐19 incidence and mortality in older people than in younger ones. This was particularly noticeable during the early phases of the pandemic when epidemiological data demonstrated the higher rates of infection and disease severity in older people (Jordan et al., 2020; Le Couteur et al., 2020; Li et al., 2020; Yang et al., 2020). Noteworthy, the COVID‐19 case fatality rates, namely the ratio of deaths to the total number of confirmed infections, was four times higher in people over 60 compared to younger ones (Santesmasses et al., 2020). In light of these age‐related differences, COVID‐19 has been also defined as “an emergent disease of aging” (Santesmasses et al., 2020, p. 1).

The enormous amount of information and news focusing on the higher risk for older adults has been considered responsible for the sharp increase worldwide in ageist messages suggesting that COVID‐19 is exclusively a disease of the senior population (Jimenez‐Sotomayor et al., 2020). A clear example of this ageist phenomenon is the appearance and the rapid rise of the Twitter hashtag #boomerremover referring to the fact that baby boomers (people born between 1946 and 1964) were more likely to be infected and die from COVID‐19 (Brooke & Jackson, 2020; Jimenez‐Sotomayor et al., 2020). Overall, this kind of ageist message not only conveys a despicable view of older people as vulnerable and helpless victims of COVID‐19 but can also lead to biased risk perception in younger people (Jimenez‐Sotomayor et al., 2020). Specifically, healthy younger individuals might perceive themselves as invulnerable to COVID‐19 and, thus, might not consider it relevant to adhere to infection prevention behaviours (Newey, 2020). This is especially true if considering that younger individuals, compared to older ones, tend to perceive themselves as more healthy (Kaleta et al., 2009). Besides, older age groups, when compared to younger age groups, perceived more risk and more frequently adhered to the guidelines (Carlucci et al., 2020). Similar results were also reported by a large US survey showing that, compared to younger people, older ones reported a higher risk of dying from COVID‐19 (Bruine de Bruin, 2020).

Given this association between COVID‐19 and being older and proposed biased risk perception in younger individuals, we aimed at evaluating whether making people under 60 actively think of their own future as an older adult would impact their evaluation of their perceived risk for COVID‐19‐related and other medical conditions with varying degrees of age‐relatedness.

## THE PRESENT STUDY

Overall, the current study aimed at contributing to the understanding of the social priming phenomenon by testing its effects on a yet uninvestigated domain: A specific health‐context, namely people's perceived likelihood of incurring various medical conditions. This is especially relevant since previous empirical investigations have focused almost exclusively on the activation of social representations, including stereotypes and personality traits, through priming to test whether this manipulation could lead to differences in social judgements and behaviours (Molden, 2014), such as racial prejudice and social exclusion. However, no previous research has ever investigated whether social priming can be an effective technique also in the health domain.

Specifically, the present study investigated whether asking individuals to actively focus on their own future as an older individual would impact their evaluation of their perceived risk for incurring during their lives in COVID‐19‐related and other medical conditions as a function of their age relatedness. Specifically, we tested our hypotheses of overestimated perceived risks on participants under 60 years old who were asked to think about and describe themselves at the age of 70 (future as an older individual) or after just 1 year (control condition) and then indicate their perceived risk of COVID‐19 (three items; e.g., testing positive for coronavirus), other medical conditions strongly (five items; e.g., Alzheimer's disease) or weakly (five items; e.g., AIDS) related to age. We specifically focused on people under 60 years old because they are more likely to have biased risk perception due to common messages conveying the idea that COVID‐19 mostly affects older‐aged people.

Based on the reviewed literature on the association between a focus on future as an older‐aged adult, loss aversion and risk conservatism (Baumeister et al., 2016; Hershfield et al., 2011; Monroe et al., 2017) as well as the social priming literature (e.g., Kawakami et al., 2003), we developed the following hypotheses:

**H1:** Thinking about one's own future as an older individual would lead to overestimating one's own perceived risk for age‐related medical conditions in one's own life.

**H2:** This effect would be more pronounced for strongly age‐related medical conditions and the new coronavirus.

Specifically, we hypothesised that thinking about one's own future as an older individual would lead to overestimating one's own perceived risk for age‐related medical conditions in one's life (H1: Hypothesis 1). This effect would be more pronounced for strongly age‐related medical conditions and the new coronavirus as opposed to weakly age‐related medical conditions, given that mass media have frequently depicted COVID‐19 as a highly age‐related medical condition (H2: Hypothesis 2).

This latter hypothesis is also supported by empirical evidence on social category priming (Kawakami et al., 2003). People primed with the “older age” category are more likely to assimilate their behaviours and beliefs to the stereotype of older individuals. For example, people presented with a photograph of an older‐aged woman and asked to describe her personality and hobbies reported more older‐congruent attitudes than participants primed with the young‐people category (Kawakami et al., 2003). Based on this literature, we hypothesised the same effect when people are “primed” to think of themselves as older people and they would thus overestimate their perceived risk of developing medical conditions with varying degrees of age‐relatedness, in particular the strongly related ones, during their life.

All these hypotheses have been tested by focusing on a group of Italian people under 60 years old. Italy represented a relevant case study since the Italian media heavily spread messages suggesting that COVID‐19 is mainly a disease of older people (e.g., Petretto & Pili, 2020). This idea has been conveyed also during the daily press conferences on the spread of COVID‐19 in Italy. Specifically, on these occasions, people from the Civil Protection Department and the National Institute of Health frequently focused on the higher risk for older people by giving data divided by age groups.

## METHOD

### Participants

One hundred and forty‐eight participants aged between 18 and 60 were recruited through Prolific Academic (www.prolific.com), an online platform used for data collection. Four of them were excluded as they did not reside in Italy during the COVID‐19 pandemic.

The remaining 144 participants were mainly men (55.6%) with a mean age of 27.72 (*SD* = 8.25, range = 18–56). The majority of respondents were in a relationship or married (56.2%) and were students or working students (55.6%); in addition, 49.3% of participants earned at least an upper secondary school diploma. The 19.4% lived in Northwest Italy, 22.9% in Northeast Italy, 27.1% in Central Italy, 19.4% in Southern Italy, and 11.1% in Insular Italy. A summary of the participants' characteristics is reported in Table 1. The study was approved by the Ethical Committee of the University of Surrey (SAGE reference number: 514292‐514283‐58510465) and was conducted in compliance with the Declaration of Helsinki ethical standards. Informed consent was obtained from all participants. There did not appear to be any risk for participants involved in the research study. All the participants were paid 1.4€ each for their time.

**TABLE 1:** Participants' sociodemographic characteristics

Sociodemographic	Frequencies	%
Gender		
Male	80	55.6
Female	64	44.4
Age (mean and standard deviation)	27.72 (8.25)	
Marital status		
Single	61	42.4
In a relationship	69	47.9
Married	12	8.3
Divorced/separated	1	0.7
Widowed	1	0.7
Education level		
Primary school	0	0
Lower secondary school	6	4.2
Upper secondary school	65	45.1
Bachelor's degree	42	29.2
Master's degree	27	18.8
Ph.D. or others	4	2.8
Employment status		
Student	63	43.8
Working student	17	11.8
Part‐time employed	9	6.3
Full‐time employed	31	21.5
Unemployed	17	11.8
Other	7	4.9
Region of residence (official statistical regions)		
Northwest Italy	28	19.4
Northeast Italy	33	22.9
Central Italy	39	27.1
Southern Italy	28	19.4
Insular Italy	16	11.1

The required sample size was calculated from an a priori power analysis for two groups by three medical conditions interaction (ANOVA: Repeated measures, within‐between interaction), using the software Gpower 3.1.9.6 (Faul et al., 2009). To detect a weak effect size ($\eta_{p}^{2}$= 0.02), with a power of 80%, significance level at 5%, a correlation among repeated measures equal .30, and nonsphericity correction ϵ equal .70, the a priori power analysis revealed a minimum sample of 144 participants.

### Materials and procedure

The research was conducted through a survey on Qualtrics®, an Internet‐based survey tool, and it was presented as “a study of health‐related lifestyles”. We applied filters to Prolific to select participants within the 18–60 age range, Italian nationals currently resident in italy. Individuals interested in participating were given a link to a survey on Qualtrics® where a brief introduction about the questions included in the survey, some instructions to complete it (e.g., read the questions carefully, be sincere), and the consent form could be accessed. All data were collected on the 13th March 2020, that is, 3 weeks after Patient One tested positive for COVID‐19 and 3 days after the extension of the lockdown to the whole of Italy. In March 2020, Italy approved severe restrictions to the entire population, with significant consequences on the citizens' vulnerability and risk perception (Brivio et al., 2020; Masiero et al., 2020; Monzani et al., 2021; Pancani et al., 2021).

After giving consent, participants were randomly assigned to either the future as an older individual (*n* = 65) or the control condition (*n* = 79). Specifically, by adapting the task developed by Packer and Chasteen (2006), people in the experimental group were asked to think about their future self at the age of 70: “This task will ask you to spend one minute thinking deeply about your own future. Imagine yourself at the age of 70. Really try to put yourself in the shoes of your future self and attempt to understand what life will be like for you then. What sorts of things will you be doing? How will you feel? What sorts of things will you think about?”. After 1 minute, participants were automatically redirected to the following page, where they were given 2 minutes to write a short narrative essay (minimum 100 characters) about what their life will be like at that time. Instructions for the control group were identical except that people were asked to imagine themselves “in one year from now.”

Then, perceived risk was assessed with 13 items asking participants to indicate their own likelihood of experiencing COVID‐19‐related conditions (three items), strongly age‐related (five items), and weakly age‐related (five items) medical conditions in their life on a 0–100 slider, with 0 meaning “It is very unlikely that it will occur to me” and 100 meaning “It is very likely that it will occur to me.” We drew the strongly and weakly age‐related medical conditions from a study on the influence of perceived age‐relatedness of medical conditions on people's comparative optimism for contracting them (Madey & Gomez, 2003). Specifically, in that study, age‐relatedness was measured by asking participants whether ageing was a likely cause in contracting 24 common medical conditions. Among these medical conditions, we selected the five items with the highest mean rating (i.e., strongly age‐related medical conditions) and the five with the lowest rating (i.e., weakly age‐related medical conditions).^1^ The order of presentation of the 13 items was randomised for each participant.

At the end of the survey, participants were asked some socio‐demographic questions and a question to establish whether they understood and remembered the future self‐manipulation. Specifically, they were asked to indicate whether the instructions asked them to think about and describe themselves: (a) at the age of 50, (b) at the age of 60, (c) at the age of 70, (d) in 1 year from now, (e) in 5 years from now, (f) in 10 years from now.

Finally, participants were debriefed and thanked. Although additional measures were collected in this study, for the sake of conciseness and clarity, we report and analyse only the measures related to participants' perceived risk in the current work.

## RESULTS

### Preliminary analyses

The experimental and the control group did not differ in either age [experimental group: *M* = 27.25, *SD* = 8.18; control group: *M* = 28.10; *SD* = 8.33; *t*(142) = 0.62, *p* = .538] or gender [experimental group: 47.7% male; control group: 62.0% male; χ^2^(1) = 2.97, *p* = .085].

According to a chi‐squared test, the large majority of participants were accurate in reporting the instruction of future‐oriented thinking [χ^2^(1) = 140.02, *p* < .001]. Specifically, 94.9% of participants in the control group and 98.5% of those in the experimental group reported the appropriate instruction. Thus, participants understood and could recall the instructions of the manipulation of future‐oriented thinking. This is important to establish that participants' focus was directed to the relevant thinking condition, thus providing the basis for an effect to occur, even at an implicit level.

Descriptive statistics of the 13 items assessing perceived risk for the overall sample as well as the two experimental groups are reported in Table 2. This table also displays the results of independent‐sample *t*‐tests assessing differences between the two groups. As shown, in our study, the highest value of perceived risk across the two experimental groups was reported for “flu” (*M* = 77.97, *SD* = 23.30), while the lowest score was for “AIDS” (*M* = 13.89, *SD* = 15.39). Moreover, while the two experimental groups did not differ in COVID‐19‐related perceived risk, perceived risk was significantly higher and yielded large effects in the experimental than in the control group for all the strongly age‐related medical conditions. Similarly, although the effects were weaker in magnitude, participants primed with thinking about their future as an older individual reported higher levels of perceived risk than the control participants for the risks of “appendicitis” and “flu,” two weakly age‐related medical conditions.

**TABLE 2:** List of the medical conditions included in the perceived risk scale: Means (and standard deviations) were reported for the entire sample and the two groups along with the results of the *t*‐tests comparing the two groups and their associated effect size (Cohen's *d*)

Medical condition	Total sample	Experimental group	Control group	t(df)	p‐value	d
COVID‐19						
Testing positive	49.71 (25.89)	51.22 (26.80)	48.47 (25.21)	0.63 (142)	.528	0.11
Being hospitalised	35.82 (23.94)	36.22 (25.74)	35.49 (22.52)	0.18 (142)	.858	0.03
Dying	18.61 (19.82)	20.02 (21.18)	17.46 (18.70)	0.77 (142)	.443	0.13
Strongly age‐related						
Osteoporosis	42.04 (29.22)	58.28 (24.19)	28.68 (26.17)	6.99 (142)	<.001	1.17
Cataracts	45.66 (29.49)	59.55 (24.87)	34.23 (28.17)	5.66 (142)	<.001	0.95
Alzheimer's disease	38.76 (27.24)	48.98 (25.14)	30.34 (26.13)	4.33 (142)	<.001	0.73
Vision loss	33.51 (25.66)	44.12 (25.09)	24.77 (22.78)	4.85 (142)	<.001	0.81
Hearing loss	37.56 (26.15)	49.60 (25.69)	27.65 (22.17)	5.43 (127.3)	<.001	0.92
Weakly age‐related						
Appendicitis	38.35 (25.94)	45.92 (26.98)	32.11 (23.44)	3.29 (142)	<.001	0.55
Alcohol dependency	18.03 (20.88)	20.18 (21.86)	16.27 (20.00)	1.12 (142)	.264	0.19
Flu	77.97 (23.30)	86.38 (17.30)	71.05 (25.35)	4.15 (137.6)	<.001	0.69
AIDS	13.89 (15.39)	16.51 (16.20)	11.73 (14.45)	1.87 (142)	.064	0.31
Sexually transmitted diseases	16.87 (25.80)	29.78 (27.24)	24.47 (24.47)	1.23 (142)	.220	0.21

*Note*: Perceived risk was rated on a 0–100 Likert scale. Degrees of freedom of the *t*‐tests conducted on “hearing loss” and “flu” were adjusted because the homogeneity assumption was not met.

### Factor structure of the PR scale

**Figure 1:**
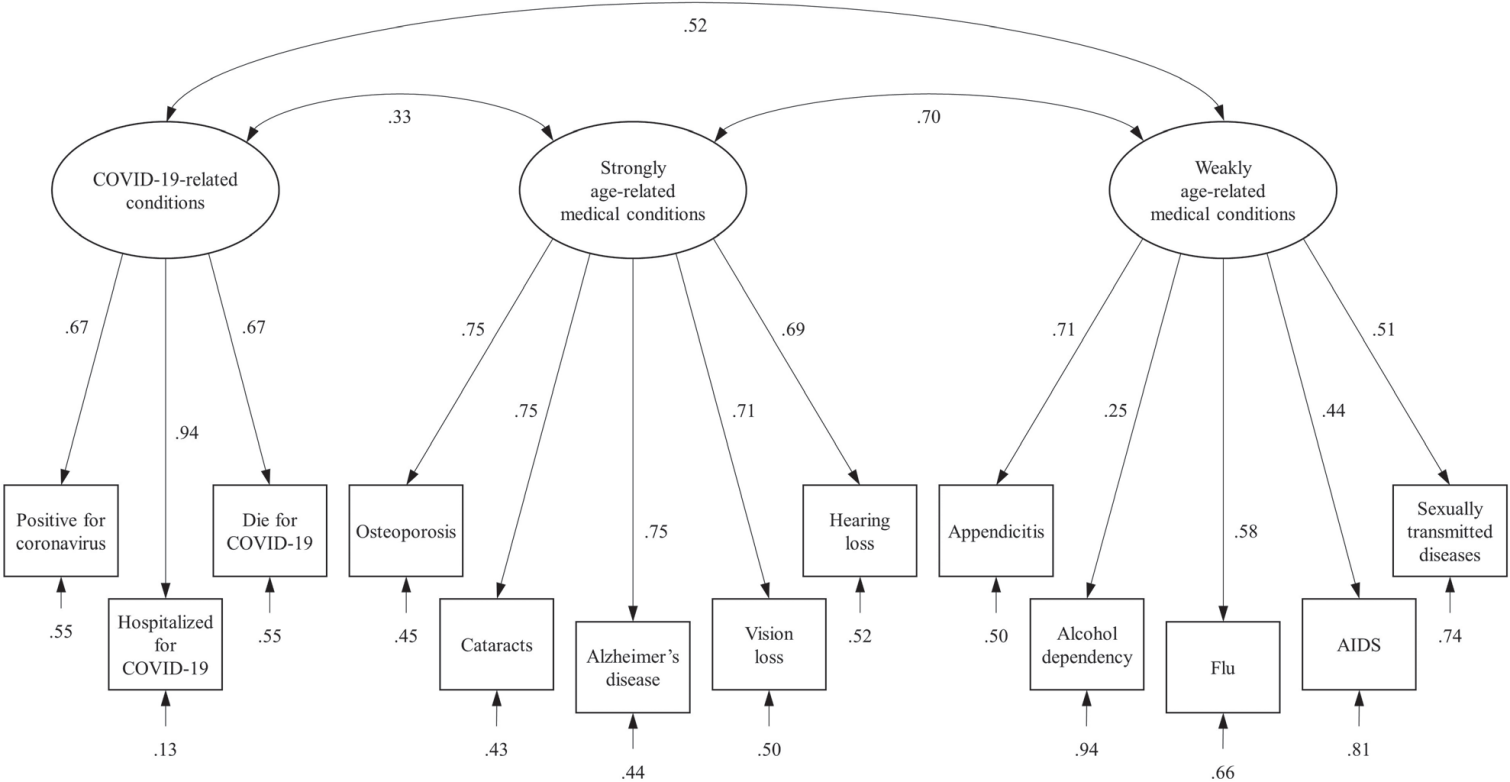
Confirmatory factor analysis yielding the hypothesised factor structure of the 13 items measuring PR.

In a first analytical step, confirmatory factor analysis was performed to test the factor structure of the 13 items measuring perceived risk, using the software Mplus version 7 (Muthén & Muthén, 2012). Specifically, we hypothesised and tested a three‐factor solution with latent dimensions measuring perceived risk respectively for: (a) COVID‐19‐related conditions (three items), (b) strongly age‐related (five items) and (c) weakly age‐related (five items) medical conditions. According to common recommendations (Brown, 2015; Kline, 2015), the fit indices were good [*χ* ^2^(62) = 100.55, *p* = .001; CFI = .935, RMSEA = .066, *p* of close fit = .135], indicating that our model fitted the data adequately. Model results are reported in Figure 1. Standardised loadings were all positive and significant, ranging between .44 and .94, with a very slow (but still significant) value for alcohol dependency (*λ* = .25). A further, one‐factor model was tested to compare the hypothesised dimensionality with an alternative one in which all the items measure a general factor of perceived risk. The results indicated a bad fit [*χ* ^2^(65) = 174.77, *p* < .001; CFI = .815, RMSEA = .108, *p* of close fit <.001] and the chi‐square difference test [Δ*χ* ^2^(3) = 74.22, *p* < .001] demonstrated that the one‐factor model was significantly worse than the three‐factor one, excluding the possibility of measuring a general factor of perceived risk.

All three factors were significantly and positively correlated. Reliability was good for factors measuring COVID‐19 conditions (α = .79) and strongly age‐related medical conditions (α = .85), whereas it was lower but still acceptable for the factor measuring weakly age‐related medical conditions (α = .62). Composite scores of the three perceive risk factors were computed as the mean of the items that loaded on them and used in the following analysis.

### Perceived risk and future self‐priming

**Figure 2:**
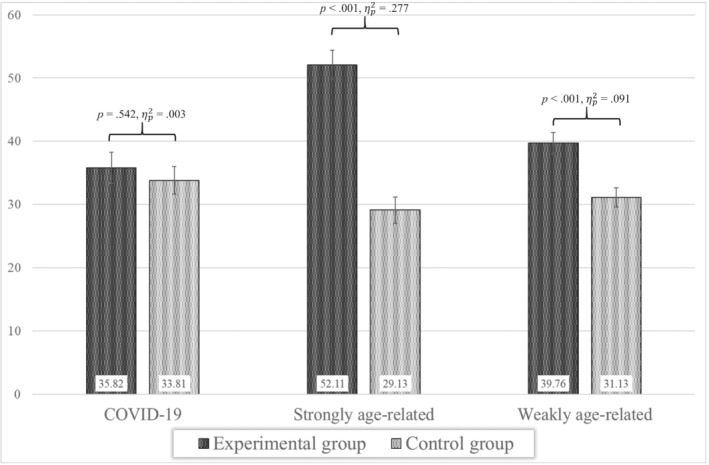
Estimated means of perceived risk for COVID‐19, strongly age‐related and weakly age‐related medical conditions in experimental and control groups with statistical significance and effect size of the Bonferroni‐adjusted post‐hoc tests. Error bars represent standard errors.

A mixed‐design ANOVA was performed to test whether ratings of perceived risk changed as a function of (a) the typology of the medical condition (three‐level, within‐subject factor: COVID‐19‐related conditions, strongly, and weakly age‐related medical conditions), (b) the experimental group (two‐level, between‐subject factor: experimental vs control group). Besides the main effects, we included a two‐way interaction term between these two independent variables to test our main hypothesis regarding differences in perceived risk. The magnitude of each effect was interpreted by considering its associated partial eta squared (i.e., $\eta_{p}^{2}$). Specifically, effects were considered weak (.01 < $\eta_{p}^{2}$ ≤ .06), moderate (.06 < $\eta_{p}^{2}$ ≤ .14), or strong ($\eta_{p}^{2}>0.14$). The results are graphically depicted in Figure 2. The ANOVA was conducted using SPSS, version 26 (IBM, 2018).

Mauchly's test indicated that the assumption of sphericity had been violated [χ^2^(2) = 14.00, *p* = .001). Thus, all degrees of freedom of within‐subject effects were adjusted using Greenhouse–Geisser estimates (ϵ = .91). The main effects of group membership [*F*(1, 142) = 25.74, *p* < .001, $\eta_{p}^{2}$ = .153] and type of medical condition [*F*(1.83, 259.48) = 7.31, *p* = .001, $\eta_{p}^{2}$ = .049] were significant. Specifically, the overall perceived risk was higher in the experimental group (*M* = 42.56, *SE* = 1.64) than in the control group (*M* = 31.36, *SE* = 1.48) and strongly age‐related medical conditions were perceived more likely to occur (*M* = 40.62, *SE* = 1.56) than both weakly age‐related (*M* = 35.44, *SE* = 1.15) and COVID‐19‐related (*M* = 34.81, *SE* = 1.64) conditions, which did not differ from one another. However, these main effects were further qualified by the significant interaction between group membership and typology of medical condition [*F*(1.83, 259.48) = 20.643, *p* < .001, $\eta_{p}^{2}$= .127].

The Bonferroni‐adjusted post‐hoc tests revealed that, compared to those in the control group, participants in the experimental group perceived themselves more likely to develop both weakly [*F*(1, 142) = 14.21, *p* < .001, $\eta_{p}^{2}$ = .091; experimental group: *M* = 39.76, *SE* = 1.70; Control: *M* = 31.13, *SE* = 1.53] and strongly [*F*(1, 142) = 54.32, *p* < .001, $\eta_{p}^{2}$ = .277; experimental group: *M* = 52.11, *SE* = 2.31; Control: *M* = 29.13, *SE* = 2.09] age‐related conditions, though the effect was larger for the latter. Conversely, participants in the experimental and control groups did not differ in perceived risk for COVID‐19‐related conditions [*F*(1, 142) = 0.37, *p* = .542, $\eta_{p}^{2}$ = .003; experimental group: *M* = 35.82, *SE* = 2.43; Control: *M* = 33.81, *SE* = 2.21].

## DISCUSSION

This work is the first attempt to test the effect of priming a future time frame on individuals' risk perception within the health context. The literature on social priming has mainly focused on the investigation of the priming's effect on social judgements and behaviours, such as stereotypes, racial prejudice and social exclusion (Molden, 2014). Specifically, while previous empirical evidence attested that the social category priming is effective in manipulating people's ageing stereotypes (Kawakami et al., 2003) and enhancing perceived risk and risk aversion in financial decisions (e.g., Hershfield et al., 2011; Monroe et al., 2017), we furthered this investigation by demonstrating that an active focus on the future as an older individual is a viable means to enhance the perception of health‐related risk. Overall, this study contributes to the social priming literature by showing that this kind of manipulation could be used to effectively change the evaluation of the personal risk of being affected by medical conditions other than manipulating social behaviours and judgements.

Our rationale for focusing on leveraging perceived risk and especially on COVID‐19‐related risk perception in people less than 60 years old was twofold. First, risk perception is among the key determinants of behavioural intentions and the adoption of healthy and precautionary behaviours. Thus, heightening people's perceived likelihood of experiencing COVID‐19 might have a beneficial effect on their adoption of precautionary measures, including mask‐wearing, hand washing, and social distancing. Second, recent empirical evidence suggests that younger people might underestimate their risk of being infected by the new coronavirus and report lower perceived risk and fewer precautionary measures than older people do (Bruine de Bruin, 2020; Carlucci et al., 2020; Newey, 2020). This biased perception might also be increased by institutional sources strongly emphasising the association between being older and the risk of COVID‐19 morbidity and mortality (Petretto & Pili, 2020) and consequent ageist messages suggesting that COVID‐19 is one of the main concerns only for older people (Jimenez‐Sotomayor et al., 2020).

Thus, building on empirical evidence showing that thinking about their future as an older individual can lead people to focus on uncertainty and increase their risk avoidance, caution and attentional focus on risks (Baumeister et al., 2016; Monroe et al., 2017), we tested whether thinking about and describing themselves at the age of 70 would be effective in fostering perceived risk for COVID‐19 as well as strongly and weakly age‐related medical conditions. We also hypothesised that this manipulation would be more effective in fostering the perceived risk of experiencing medical conditions, such as COVID‐19 and age‐related diseases, that are generally depicted as affecting mainly older people.

While our main hypothesis regarding the leveraging effect of thinking about the future as an older individual was supported overall, as shown by the significant and large main effect of the thinking condition to which participants were assigned, we did not find evidence supporting the influence of our manipulation on the risk estimates for COVID‐19‐related conditions.

Specifically, participants in the experimental group reported higher perceived risk than people in the control group. Overall, this result suggests that this kind of manipulation could have practical value in fostering people's risk estimates of being affected by chronic and acute diseases typically associated with ageing (e.g., osteoporosis). This could be especially relevant if generalised to the primary and secondary prevention of lifestyle diseases, namely illnesses linked with and caused by individuals life‐style (e.g., cancer, cardiovascular and respiratory diseases). Overall, these results attested that social category priming can be also used to manipulate personal health‐related perceptions and not just social behaviours and judgements. Future research should assess whether increasing people's perceived vulnerability to this kind of medical condition through actively thinking about their future as an elderly individual may lead to a subsequent higher adoption rate of effective preventive and precautionary behaviours. For instance, it will be relevant to assess whether people within prevention and rehabilitation programmes for cardiovascular diseases, if primed with thinking about their future as older people, would perceive a higher risk and hence adopt a healthier lifestyle.

This rationale also underlaid our main focus on the leverage of personal risk estimation about COVID‐19. However, contrary to our hypothesis, we did not find significant differences between the two experimental groups in COVID‐19‐related perceived risk. In contrast, we found that people primed with thinking about their older age reported higher perceived risk for both weakly and strongly age‐related conditions. The effect size of these differences was only moderate for the former but greater for medical conditions being commonly depicted as mainly affecting older adults. Among other potential explanations, the result of a lack of influence of our manipulation on perceived risk for COVID‐19 might be caused by a possible generalised effect involving the estimation of the risk of being infected, having severe consequences, and eventually dying from COVID‐19. Specifically, even if a direct comparison with objective data is not possible, due to the scale format used in this study, an approximate comparison between the actual, scientific values and participants' estimates indicates that risk is overestimated more for COVID‐19‐related conditions than for the other medical conditions. Thus, we might assume that our manipulation might be ineffective for COVID‐19‐related risk because of this presumable “ceiling” effect. A high overestimation affecting individuals' perception of their COVID‐19‐related risk would be consistent with empirical evidence showing that people are more likely to perceive the risk of a phenomenon as higher when they perceived it is out of their personal control, has possible fatal outcomes, and is novel (Slovic, 1987). Interestingly, all these characteristics are among the main distinctive features of this pandemic as well as other previous epidemics. The affect heuristic and negative emotion strongly associated with the new pandemic can also have a relevant role in inflating people's estimation of their related risk (Byrne et al., 2020).

While our results suggested that Italian people, under 60, were appropriately concerned about the serious hazard in the very early phases of the ongoing pandemic, this might seem in contrast with other empirical evidence showing that younger people were less adherent to recommended infection prevention behaviours (Carlucci et al., 2020; Newey, 2020). However, highly negative emotion‐charged messages and fear appeals aimed at promoting risk perception may be also counter‐productive for adopting preventive behaviours because people may aim at mitigating the fear even through denial or avoidance instead of showing functional conducts (Lin, 2020). Thus, as suggested by Hamilton et al. (2020), during the ongoing pandemic effective infection prevention behaviours should be promoted within the general population by implementing health messages that highlight risk perception while providing coping information to enhance peoples' self‐efficacy. The results of Roma et al. (2020) also highlight the moderation role of risk perception on the relationship between perceived efficacy of protective guidelines and compliance with protective measures. This suggestion is also coherent with one of the main paradigms of crisis communication stating that when people are concerned about a severe hazard, they should be helped in bearing their fear and guided to the choice of effective precautionary measures (Sandman, 2006).

## LIMITATIONS AND FUTURE DIRECTIONS

Despite meaningful results for the social priming and preventive behaviour literature, the present study is not devoid of limitations. The main limitation of this study is that we did not measure the expectancies about the arrival of a vaccine for the different conditions. In particular, it is possible that some participants expected an efficacious vaccine within a few years. However, we believe that this did not affect our conclusions, because even if in the experimental condition participants thought of themselves in the future (when they will be 70), the questions related to the risk for COVID were referred to their course of life in general, thus, potentially, not even earlier than in 35 or 40 years. This is corroborated by the relatively high estimates provided by the participants for the COVID‐19‐related risks despite the possibility of being vaccinated.

Similarly, it should be considered that the study has been conducted during the first Italian lockdown and wave of the COVID‐19 pandemic. This situation fluctuates over time and it is characterised by high uncertainty at both individual and societal levels. For example, at that time it was unsure how the pandemic would evolve in the near future, in terms of potential cyclic waves and coronavirus variants, as well as long‐term consequences of COVID‐19 (i.e., “long COVID,” Huang et al., 2021; Venkatesan, 2021; Venturelli et al., 2021). Specifically, the view of the COVID‐19 as an emergent disease of ageing, that was frequently conveyed during the first wave, has been challenged by new empirical evidence and media focus on the increased risk for people under 60 of having deleterious consequences from COVID‐19. It now appears that younger people are more likely to be infected by new coronavirus variants and long COVID (Ludvigsson, 2020; Sugden & Colchester, 2021). Replication studies should be performed to assess whether the results of this paper would hold also in later stages of the COVID‐19 pandemic. Along the same lines, the target age of the priming manipulation could be changed as well. Specifically, by considering the increased risk of being infected and having long‐term consequences from COVID‐19 at younger ages, future research might evaluate whether asking people, in the experimental condition to actively think of and describe themselves as middle‐aged adults, would have a differential effect on their perceived risk for COVID‐19 and other medical conditions varying for age‐relatedness.

Finally, the present study examined a sample made up of individuals who are on average young 27.72 (*SD* = 8.25, range = 18–56). This was necessary to effectively manipulate participants' focus on a future time frame. For example, in the experimental condition, participants aged 60, the age limit for the present study, had to think of themselves at the age of 70, thus in 10 years. Recruiting a sample with a different age range (e.g., 60–80 years) could have undermined the effectiveness of the experimental manipulation by narrowing the gap between the current participant's age and the age of their future self to focus on in the experimental condition. Future research should thus address the issue of age‐related individual differences by means of a finer‐grained experimental manipulation such as one that relies on age‐morphing and virtual reality as in Hershfield et al.'s (2011) study.

## CONCLUSIONS

This study showed that asking people to actively engage with their future (either proximal, in a year, or distal, at the age of 70) elicits relatively high perceived risk within the health domain, thus adding to the literature on social priming that has only considered this type of priming effect with reference to monetary risks (e.g., Hershfield et al., 2011; Monroe et al., 2017). The results of the present study showed that prompting participants with a focus on their distal future as an older person, as opposed to their proximal future, enhanced their risk perception for age‐related conditions. Further, we highlighted a boundary condition not yet investigated in previous social priming research: Our manipulation was ineffective in addressing COVID‐19‐related risk perception, a finding that we interpreted as a result of the current salience of COVID‐19‐related conditions.

These results could be used to tailor communication strategies about health information and screening programmes by addressing the temporal dimension of perceived risk (e.g., Hall & Fong, 2007). For example, it has been shown that an unhealthy behaviour such as smoking is associated with inadequate risk perceptions as shown by estimates of disease onset that are delayed in the future (Pancani & Rusconi, 2018). In this sense, shifting individuals' thinking time frame to the future could facilitate more accurate risk perceptions and related engagement in health behaviours. Specifically, further research is needed to understand whether messages highlighting the late‐life health repercussions could be valid interventions in clinical practice and preventive and rehabilitation programmes to improve perceived risk while counteracting excessive time discounting of potential future outcomes and risky behaviours.

## Endnotes

^1^
We excluded “measles” because, contrary to the other infections, in many cases people who have been infected will then acquire a lifelong immunity to it.
